# Three Cases of Pseudo-Meigs' Syndrome Secondary to Ovarian Metastases from Colorectal Cancer

**DOI:** 10.1155/2017/5235368

**Published:** 2017-03-08

**Authors:** Yuta Yamamoto, Yusuke Miyagawa, Takehito Ehara, Makoto Koyama, Satoshi Nakamura, Daisuke Takeuchi, Futoshi Muranaka, Masato Kitazawa, Shinichi Miyagawa

**Affiliations:** Department of Gastroenterological Surgery, Shinshu University School of Medicine, Matsumoto, Japan

## Abstract

Pseudo-Meigs' syndrome is used to describe cases of ascites and/or pleural effusion associated with ovarian neoplasms other than benign tumors, which improve after removal of the ovarian lesion. We present three cases of pseudo-Meigs' syndrome secondary to ovarian metastasis from colorectal cancer. In case 1, the patient has severe dyspnea and hypoxia due to massive right pleural effusion; therefore preoperative thoracic drainage was needed. In case 2, the patient needed paracentesis every two or three days to improve abdominal distension. After two courses of 5-fluorouracil, levofolinate, and oxaliplatin (mFOLFOX6), her ascites could be controlled by diuretics without aspiration and her general condition improved. Then she underwent operation. In case 3, the patient developed a massive pleural effusion and ascites coincident with a rapid enlargement of ovarian tumor after resection and adjuvant chemotherapy for rectal cancer. In all cases, pleural effusions and/or ascites resolved and general conditions and daily activities of the patients improved after oophorectomy. They are all currently in good health without recurrence of pleural effusion or ascites. In patients with suspected pseudo-Meigs' syndrome secondary to ovarian metastasis of colorectal cancer, operation including oophorectomy may reduce pleural effusions and/or ascites and improve the general condition.

## 1. Introduction

Meigs and Cass reported 7 cases of nonmalignant ascites and/or pleural effusion associated with benign ovarian tumors that improved after removal of the ovarian lesion [1]. Thereafter, this syndrome was called Meigs' syndrome [2]. In contrast, pseudo-Meigs' syndrome is used to describe cases of ovarian neoplasms other than benign primary tumors that cause the same syndrome [3, 4]. In this article, we present three cases of pseudo-Meigs' syndrome secondary to ovarian metastasis from colorectal cancer.

## 2. Case Presentation

### 2.1. Case  1

**Figure 1:**
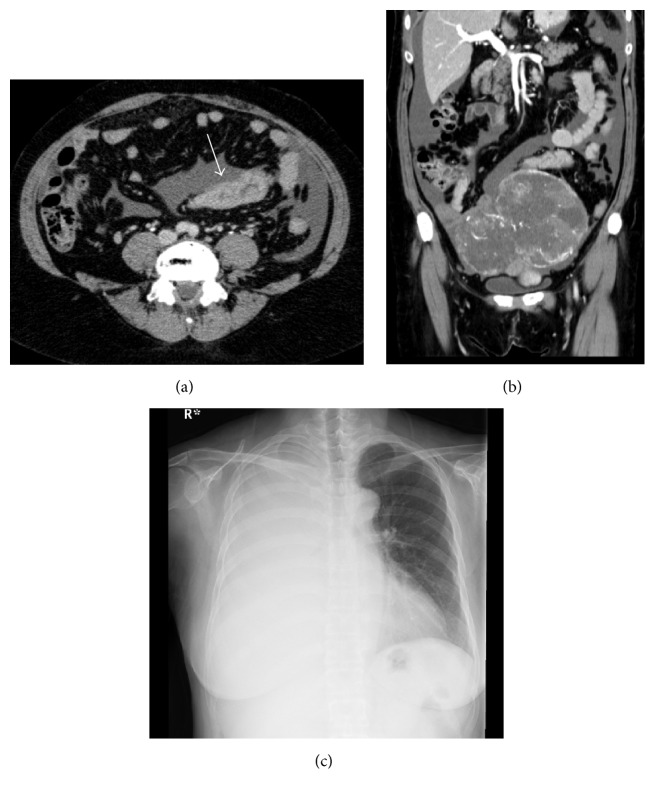
Case  1. Contrast-enhanced CT scan shows wall thickening of the sigmoid colon with enhancement (arrow; (a) axial plane) and a well-circumscribed and heterogeneously enhanced tumor of the ovary ((b) coronal plane). Chest X-ray shows a massive pleural effusion in right thorax (c).

**Figure 2:**
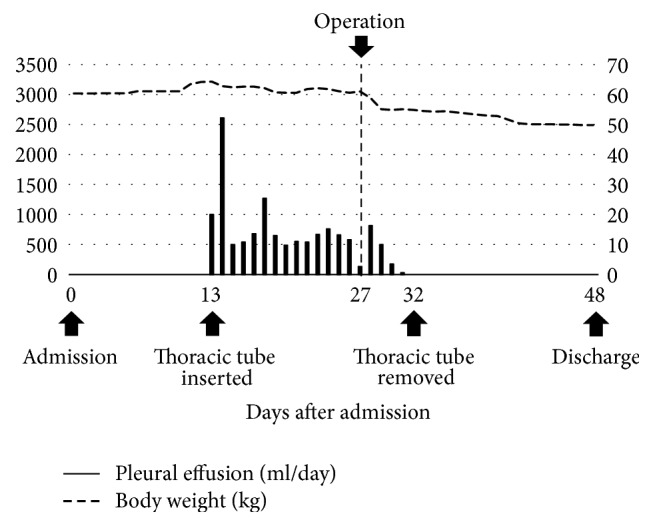
Changes in pleural effusion drainage during the perioperative period in case 1. The bars show amounts of pleural effusion drained from the right thorax each day throughout the clinical course.

**Figure 3:**
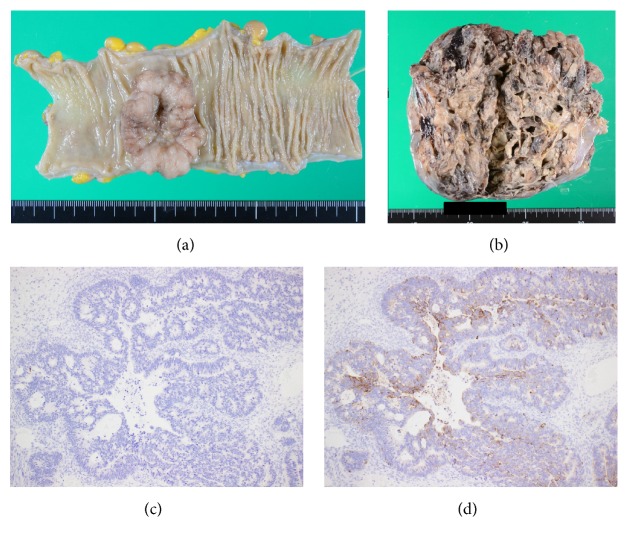
Macroscopic findings of resected specimen from case 1. An ulcerative tumor in the sigmoid colon (a). The removed ovarian tumors weighed 1,102 g (left, (b)) and 490 g (right). The cut surface of the ovarian tumor shows focally villous appearance with cystic formation. Immunophenotype of a cytokeratin 7− (c) and cytokeratin 20+ (d) was seen in the ovarian tumor.

A 48-year-old woman was admitted to the local clinic with vomiting. She was diagnosed with bilateral ovarian tumors. Right pleural effusion rapidly increased. She had severe dyspnea and was transferred to our hospital. Her abdomen was distended, and an abdominal mass was palpated. Laboratory tests showed anemia and elevated serum tumor marker levels (CEA: 24.5 ng/dL, CA19-9: 56.2 U/mL, and CA125: 606.1 U/mL). A chest X-ray showed a right pleural effusion. The cytology of the pleural effusion was class II. Colonoscopy identified a type 2 tumor in the sigmoid colon and a biopsy specimen from the tumor histopathologically showed a moderately differentiated adenocarcinoma. Contrast-enhanced CT scan displayed wall thickening of sigmoid colon and a huge intrapelvic tumor (Figures 1(a) and 1(b)). Her dyspnea worsened due to increased right pleural effusion (Figure 1(c)). Thoracic drainage tube was inserted and 500–1000 mL of pleural effusion was removed each day (Figure 2). The patient underwent a sigmoid colectomy, bilateral oophorectomy, and sigmoid colostomy. At operation, there was no evidence of peritoneal dissemination and peritoneal cytology was class II. Regarding the resected specimen, the ovarian tumors were well-circumscribed. In the sigmoid colon, a type 2 tumor, 55 × 35 mm in diameter, was observed (Figure 3(a)). Histopathological examination showed that both the ovarian and colonic lesions were composed of well-differentiated adenocarcinoma. Immunophenotype of a cytokeratin 7− (Figure 3(c)) and cytokeratin 20+ (Figure 3(d)) was seen in the ovarian tumors and they were considered to be metastasis of sigmoid colon cancer. The postoperative course was uneventful, and the thoracic tube was removed on the 5th postoperative day (POD). She was discharged on the 21st POD. After surgery, 5-fluorouracil (5-FU), leucovorin, and oxaliplatin (mFOLFOX) were administered every 2 weeks for 6 months as adjuvant chemotherapy. And 17 months after surgery, tumor markers were within the normal range and CT showed no sign of tumor growth or recurrent pleural effusion or ascites.

### 2.2. Case  2

**Figure 4:**
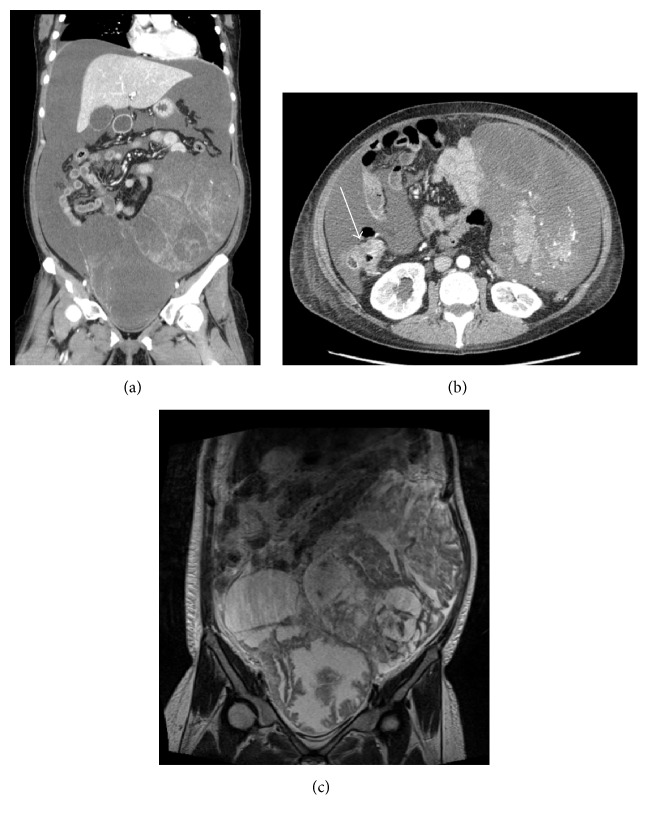
Case  2. Contrast-enhanced CT scan shows a well-circumscribed and heterogeneously enhanced tumor of the ovary ((a) coronal plane) and wall thickening of the ascending colon with enhancement (arrow; (b) axial plane). MRI T2 shows cystic masses in the pelvis ((c) coronal plane).

**Figure 5:**
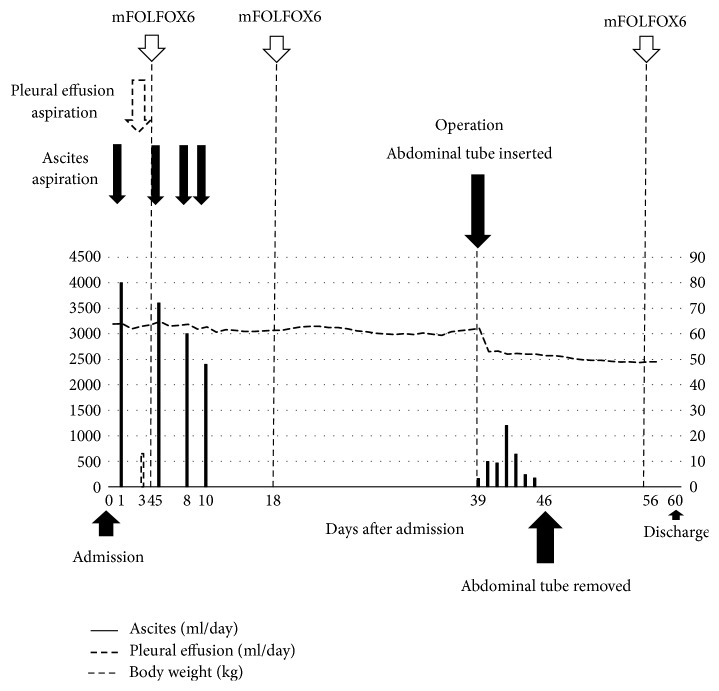
Changes in ascites and pleural effusion drainage in the perioperative period in case 2. The bars show amounts of ascites (solid) that was aspirated each day, and the broken line shows the amounts of pleural effusion drained each day throughout the clinical course.

A 33-year-old woman was admitted to previous hospital with constipation and abdominal distension, and an intrapelvic tumor was identified. Colonoscopy and CT scan revealed ascending colon cancer, bilateral ovarian tumors, and peritoneal dissemination. She needed repeated paracentesis to improve dyspnea and severe abdominal distension. She was then referred to our hospital. Laboratory tests showed anemia, malnutrition, and elevated levels of tumor markers (CEA: 5.8 ng/mL, CA19-9: 119.4 U/mL, and CA-125: 1400.7 U/mL). Colonoscopy showed a type 3 tumor in the ascending colon, and a biopsy specimen revealed well-differentiated adenocarcinoma. CT scan displayed bilateral pleural effusion, massive ascites, wall thickening of the ascending colon with enhancement, and bilateral cystic ovarian tumors that were about 20 cm in diameter (Figures 4(a) and 4(b)). There were three masses in the liver, suggesting metastases. Magnetic resonance imaging (MRI) T2 showed polycystic tumors in the bilateral ovaries (Figure 4(c)). The patient needed paracentesis every two or three days to improve abdominal distension (Figure 5). After two courses of preoperative chemotherapy with mFOLFOX6, ascites was reduced and controlled by diuretics without aspiration, and her general condition improved. On the 21st day after last chemotherapy, primary tumor shrinkage caused intestinal obstruction. And the patient underwent a palliative operation including bilateral oophorectomy and right hemicolectomy. There were 3 L of pale yellow serous ascites in the abdominal cavity, and peritoneal cytology was class V with malignant cells. Histopathological examination of the resected specimen showed that both the ovarian and colonic lesions were composed of well-differentiated adenocarcinoma. Immunophenotype of a cytokeratin 7−/20+ was seen in the ovarian tumors and they were considered to be metastasis of sigmoid colon cancer. KRAS mutation testing of the tumor revealed the KRAS G12D mutation. The postoperative course was uneventful, and the abdominal drainage tube was removed on the 7th POD. She received mFOLFOX6 chemotherapy on the 17th POD, and she was discharged on the 21st POD. In spite of 6 courses of mFOLFOX after surgery, abdominal CT showed growth of the hepatic metastases. Then her treatment regimen was changed to irinotecan and S-1 (IRIS) plus bevacizumab and to 5-fluorouracil, leucovorin, and irinotecan (FOLFIRI) plus ramucirumab. Finally, she is receiving outpatient chemotherapy of regorafenib without evidence of recurrent pleural effusion or ascites for 22 months after surgery.

### 2.3. Case  3

**Figure 6:**
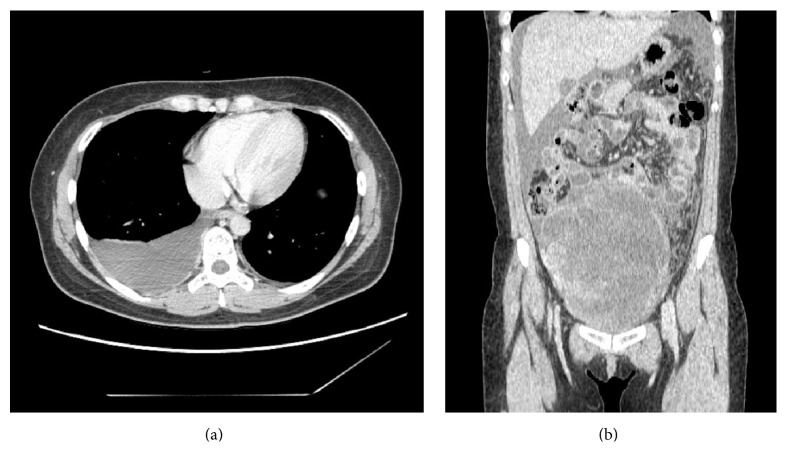
Contrast-enhanced CT scan in case 3 shows pleural effusion in the right thorax ((a) axial plane) and ascites along with a well-circumscribed and heterogeneously enhanced tumor of the rt. ovary ((b) coronal plane).

**Figure 7:**
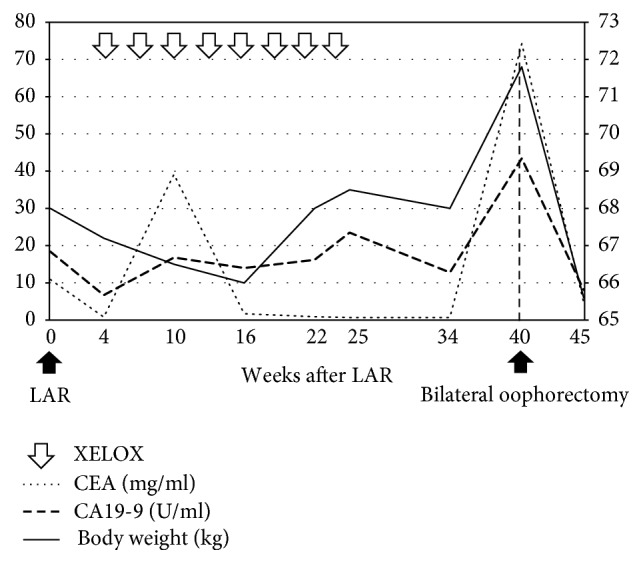
Changes in serum levels of tumor markers and body weight after primary colectomy and chemotherapy in case 3. LAR, lower anterior resection; XELOX, capecitabine and oxaliplatin.

A 27-year-old woman was diagnosed with rectal cancer and underwent low anterior resection with lymphadenectomy, and R0 operation was achieved. Histopathological examination of the specimen revealed type 2 well-differentiated adenocarcinoma that was staged T4N2M0 and pathological stage IIIc according to the International Union Against Cancer TNM Classification of Malignant Tumors (7th Edition). KRAS mutation testing revealed the KRAS Q61H mutation. She underwent transvaginal oocyte retrieval from bilateral ovaries due to her hope for pregnancy. Subsequently, she received 8 courses of adjuvant chemotherapy with capecitabine and oxaliplatin (XELOX) for 6 months. After the therapy, follow-up CT scan showed no evidence of recurrence of rectal carcinoma. However, 3 months later, the patient felt abdominal distension, and CT scan revealed an ovarian tumor and right pleural effusion (Figure 6). Laboratory tests showed anemia, malnutrition, and elevated levels of tumor markers (CEA: 74.2 ng/mL, CA19-9: 43.4 U/mL, and CA-125: 1037.4 U/mL). The patient underwent bilateral oophorectomy, and operative finding showed huge encapsulated tumor of right ovary and no evidence of peritoneal dissemination. After operation, the ascites and pleural effusion resolved (Figure 7). She received chemotherapy of 2 courses of irinotecan and S-1 (IRIS) and 7 courses of IRIS plus bevacizumab for 5 months. Now she is without sign of tumor growth or evidence of recurrent pleural effusion or ascites for 12 months after oophorectomy.

Clinical and histological findings are summarized in Table 1.

## 3. Discussion

Ovarian metastasis from colorectal cancer is not commonly encountered by oncologists. Women with colon cancer reportedly developed metastasis to the ovary in 4.0% to 30.8% of cases [5, 6]. Metastatic neoplasms comprised 7.0% of frankly malignant ovarian tumors [7], 17.1–51.7% of which were secondary to colorectal cancer [7–10].

Former studies suggested that there are three possible routes of metastasis for colon cancer to the ovary: the lymphatic vessels, blood vessels, and peritoneal dissemination. Sato et al. [11] and Tomiki et al. [12] supported the theory of metastasis through blood vessels due to the lack of lymphatic connection between colon and ovary, but Samanth and Black 3rd [13] and Lemming [14] have supported a lymphatic metastasis theory because of the pathological features of colon cancer lymphatic invasion in cases with ovarian metastasis. On the other hand, Terada et al. [15] reported that multivariate analysis identified age (premenopausal), morphological abnormalities of the ovary, depth of tumor invasion, and peritoneal metastasis as factors significantly associated with ovarian metastasis from colorectal cancer. In the present cases, patients 1 and 3 were premenopausal, and patient 2 had a history of heterotopic endometriosis. Additionally, patient 3 took ovulatory drugs and underwent transvaginal oocyte retrieval from bilateral ovaries before adjuvant chemotherapy. Taken together with the previous reports, the present cases indicate that a rupture of surface layer of the ovary, such as ovulation and heterotopic endometriosis, triggers ovarian metastasis from colorectal cancer associated with peritoneal dissemination.

With regard to the etiology of ascites, Graffner et al. [16] proposed that there is a discrepancy between the arterial supply to a large tumor mass and that its venous and lymphatic drainage could lead to stromal edema and transudation. Rhoads and Terrell [2] reported the possibility that pressure on the lymphatics in the tumor itself may cause fluid escape through the superficial lymphatics. The etiology of pleural effusion is also unclear. The transfer of ascitic fluid via transdiaphragmatic lymphatic channels is the current prevailing theory [17, 18]. Pleural paracentesis may be useful to exclude carcinomatous pleurisy.

It may be difficult to distinguish between metastasis to the ovary from colon cancer and primary ovarian carcinoma. Loy et al. [19] reported that cytokeratin immunostaining can help distinguish them; a cytokeratin 7−/cytokeratin 20+ immunophenotype was seen in 94% of the metastatic colonic tumors, 5% of the mucinous carcinomas, and none of the endometrioid or serous tumors. In the present cases, we concluded that they were metastatic colon cancer to the ovary due to their cytokeratin immunophenotype.

Patients with metastatic colon cancer to the ovary are known to have poor prognoses. Terada et al. [15] reported that the 5-year survival rate of all patients with metastases to the ovary was 29.1%, and no patients with distant metastasis to sites other than ovary survived for 5 years. However, if the metastasis was limited to the ovary, the 5-year survival rate improved up to 67.5% [15]. Relatedly, a surgical approach to metastatic colon cancer to the ovary is often recommended. Chung et al. [20] reported that surgical resection may be beneficial to selected patients with metastatic colon cancer to the ovary when metastasis is limited to the pelvis. Lee et al. [21] claimed that oophorectomy significantly prolonged survival in colorectal cancer patients with metastasis to the ovary. McCormick et al. [22] argued that optimal cytoreduction (residual disease ≤ 1 cm in maximal diameter) is associated with prolonged progression-free survival and overall survival in both patients with localized ovarian metastases and widespread metastases of colon cancer. However, these results are still controversial because they were analyzed retrospectively.

## 4. Conclusions

In the present report, after operation, including oophorectomy, patients were discharged from our hospital without evidence of recurrent pleural effusion or ascites. When the patients with malignant ovarian neoplasms suffer from ascites and/or pleural effusion, it is essential to rule out pleural dissemination and carcinomatous peritonitis, make a diagnosis of pseudo-Meigs' syndrome, and consider operations including oophorectomy, which possibly reduce pleural effusion and/or ascites and improve the general condition of the patient. Further studies are warranted to elucidate etiology and efficacy of palliative operation for pseudo-Meigs' syndrome.

## Figures and Tables

**Table 1:** Patients' characteristics.

Case	1	2	3
Age (years)	48	33	27
Primary site	Sigmoid colon	Ascending colon	rectum
Onset of PMS	Synchronous	Synchronous	Metachronous
Site of pleural effusion	Right	Right	Right
Macroscopic type	Type 2	Type 3	Type 2
Diameter of primary tumor (mm)	55 × 35	35 × 20	50 × 40
Histological type	Moderately well differentiated	Well differentiated	Well differentiated
Depth of invasion	SE	SE	SE
Lymph node metastasis^*∗*^	N1	N0	N2
Other metastases	Bilateral ovaries	Bilateral ovaries, liver, peritoneal dissemination	Right ovary
Diameter of ovarian tumor (mm)	75 (right) 127 (left)	176 (right) 180 (left)	181 (right)
KRAS mutation	Wild-type	G12D	Q61H
Surgical procedure	Colectomy + bilateral oophorectomy	Colectomy + bilateral oophorectomy	1st LAR, 2nd bilateral oophorectomy
Prognosis	AWNED, 17 months after Ox	AWD, 22 months after Ox	AWNED, 12 months after Ox

^*∗*^According to International Union Against Cancer TNM Classification of Malignant Tumors (7th Edition).

LAR, low anterior resection; AWNED, alive with no evidence of disease; AWD, alive with disease; Ox, oophorectomy.

## Abbreviations

AWD: Alive with disease
AWNED: Alive with no evidence of disease
CA125: Cancer antigen 125
CA19-9: Carbohydrate antigen 19-9
CEA: Carcinoembryonic antigen
CT: Computed tomography
FOLFIRI: 5-Fluorouracil, leucovorin, and irinotecan
IRIS: Irinotecan and S-1
L: Left ovary
LAR: Lower anterior resection
mFOLFOX6: 5-Fluorouracil, levofolinate, and oxaliplatin
MRI: Magnetic resonance imaging
POD: Postoperative day
R: Right ovary
XELOX: Capecitabine and oxaliplatin.

## References

[1] Meigs J. V., Cass J. W. (1937). Fibroma of the ovary with ascites and hydrothorax. *American Journal of Obstetrics and Gynecology*.

[2] Rhoads J. E., Terrell A. W. (1937). Ovarian fibroma with ascites and hydrothorax (meigs's syndrome): report of a case. *Journal of the American Medical Association*.

[3] Meigs J. V. (1954). Pelvic tumors other than fibromas of the ovary with ascites and hydrothorax. *Obstetrics and Gynecology*.

[4] Ryan R. J. (1972). PseudoMeigs syndrome. Associated with metastatic cancer of ovary. *New York State Journal of Medicine*.

[5] Fujiwara K., Ohishi Y., Koike H., Shirafuji H., Modest E. J., Kataoka S. (1995). Clinical implications of metastases to the ovary. *Gynecologic Oncology*.

[6] Kurman R. (2002). *Blaustein's Pathology of the Female Genital Tract*.

[7] Mazur M. T., Hsueh S., Gersell D. J. (1984). Metastases to the female genital tract. Analysis of 325 cases. *Cancer*.

[8] Moore R. G., Chung M., Granai C. O., Gajewski W., Steinhoff M. M. (2004). Incidence of metastasis to the ovaries from nongenital tract primary tumors. *Gynecologic Oncology*.

[9] Ulbright T. M., Roth L. M., Stehman F. B. (1984). Secondary ovarian neoplasia. A clinicopathologic study of 35 cases. *Cancer*.

[10] Yazigi R., Sandstad J. (1989). Ovarian involvement in extragenital cancer. *Gynecologic Oncology*.

[11] Sato T., Kamano T., Uchida T., Kanno T., Sato T., Sakakibara N. (1990). Clinical study of ovarian metastasis from the colorectal cancer. *Nihon Daicho Komonbyo Gakkai Zasshi*.

[12] Tomiki Y., Kamano T., Kunii Y. (2002). Risk factors of ovarian metastasis from colorectal cancer by using multivariate analysis. *Japanese Journal of Gastroenterological Surgery*.

[13] Samanth K. K., Black 3rd W. C. (1970). Benign ovarian stromal tumors associated with free peritoneal fluid. *American Journal of Obstetrics and Gynecology*.

[14] Lemming R. (1960). Meigs' syndrome and pathogenesis of pleurisy and polyserositis. *Acta Medica Scandinavica*.

[15] Terada S., Suzuki N., Uchide K., Akasofu K. (1992). Uterine leiomyoma associated with ascites and hydrothorax. *Gynecologic and Obstetric Investigation*.

[16] Graffner H. O. L., Alm P. O. A., Oscarson J. E. A. (1983). Prophylactic oophorectomy in colorectal carcinoma. *The American Journal of Surgery*.

[17] Birnkrant A., Sampson J., Sugarbaker P. H. (1986). Ovarian metastasis from colorectal cancer. *Diseases of the Colon & Rectum*.

[18] Yamaguchi T., Urakawa T., Nakamoto M. (1989). Four cases of ovarian metastases from colo-rectal cancer. *The Japanese Journal of Gastroenterological Surgery*.

[19] Loy T. S., Calaluce R. D., Keeney G. L. (1996). Cytokeratin immunostaining in differentiating primary ovarian carcinoma from metastatic colonic adenocarcinoma. *Modern Pathology*.

[20] Chung T.-S., Chang H. J., Jung K. H. (2009). Role of surgery in the treatment of ovarian metastases from colorectal cancer. *Journal of Surgical Oncology*.

[21] Lee S. J., Lee J., Lim H. Y. (2010). Survival benefit from ovarian metastatectomy in colorectal cancer patients with ovarian metastasis: a retrospective analysis. *Cancer Chemotherapy and Pharmacology*.

[22] McCormick C. C., Giuntoli R. L., Gardner G. J. (2007). The role of cytoreductive surgery for colon cancer metastatic to the ovary. *Gynecologic Oncology*.

